# (1*R*,2*S*)-*N*,*N*′-(1,2-Dihydroxy­ethyl­ene)diformamide

**DOI:** 10.1107/S1600536808036209

**Published:** 2008-11-13

**Authors:** Amir Taheri, Sayed Mojtaba Moosavi

**Affiliations:** a Department of Chemistry, Imam Hossein University, Tehran, Iran

## Abstract

The asymmetric unit of the title compound, C_4_H_8_N_2_O_4_, contains one half-mol­ecule which is completed *via* a crystallographic inversion centre. In the crystal structure, mol­ecules are arranged in undulating layers parallel to (001). Inter­molecular N—H⋯O and O—H⋯O hydrogen bonds consolidate this arrangement.

## Related literature

The title compound has been synthesized as a by-product of a procedure described by Sidney *et al.* (1965 ▶) and Ferguson (1968*a* ▶,*b* ▶). For related literature regarding the synthesis, see: Mitsch (1965 ▶). For the application of the inter­mediates, see: Ramakrishnan *et al.* (1990 ▶); Vedachalam *et al.* (1991 ▶). For bond-length data, see: Allen *et al.* (1987 ▶).
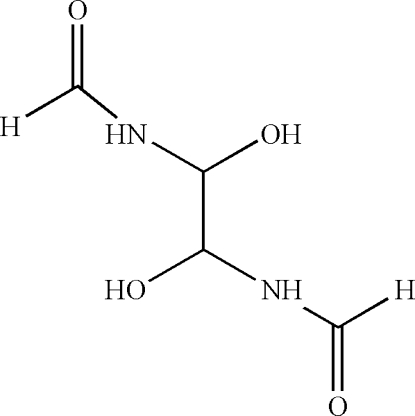


## Experimental

### 

#### Crystal data

C_4_H_8_N_2_O_4_
*M* *_r_* = 148.12
Orthorhombic, 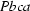

*a* = 6.5065 (11) Å
*b* = 7.2634 (12) Å
*c* = 12.772 (2) Å
*V* = 603.59 (17) Å^3^
*Z* = 4
Mo *K*α radiation
μ = 0.15 mm^−1^
*T* = 120 (2) K
0.20 × 0.20 × 0.15 mm

#### Data collection

Bruker SMART 1000 CCD area-detector diffractometer
Absorption correction: none
5931 measured reflections
796 independent reflections
662 reflections with *I* > 2σ(*I*)
*R*_int_ = 0.031

#### Refinement

*R*[*F* ^2^ > 2σ(*F* ^2^)] = 0.042
*wR*(*F* ^2^) = 0.107
*S* = 1.00
796 reflections
46 parameters
H-atom parameters constrained
Δρ_max_ = 0.41 e Å^−3^
Δρ_min_ = −0.24 e Å^−3^

### 

Data collection: *SMART* (Bruker, 1998 ▶); cell refinement: *SAINT-Plus* (Bruker, 1998 ▶); data reduction: *SAINT-Plus*; program(s) used to solve structure: *SHELXTL* (Sheldrick, 2008 ▶); program(s) used to refine structure: *SHELXTL*; molecular graphics: *SHELXTL*; software used to prepare material for publication: *SHELXTL*.

## Supplementary Material

Crystal structure: contains datablocks I, global. DOI: 10.1107/S1600536808036209/wm2200sup1.cif

Structure factors: contains datablocks I. DOI: 10.1107/S1600536808036209/wm2200Isup2.hkl

Additional supplementary materials:  crystallographic information; 3D view; checkCIF report

## Figures and Tables

**Table 1:** Hydrogen-bond geometry (Å, °)

*D*—H⋯*A*	*D*—H	H⋯*A*	*D*⋯*A*	*D*—H⋯*A*
N1—H1*N*⋯O1^i^	0.88	2.04	2.9093 (16)	170
O1—H1*O*⋯O2^ii^	0.86	1.81	2.6740 (14)	175

Symmetry codes: (i) 
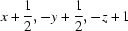
; (ii) 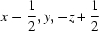.

**Fig. 1.**
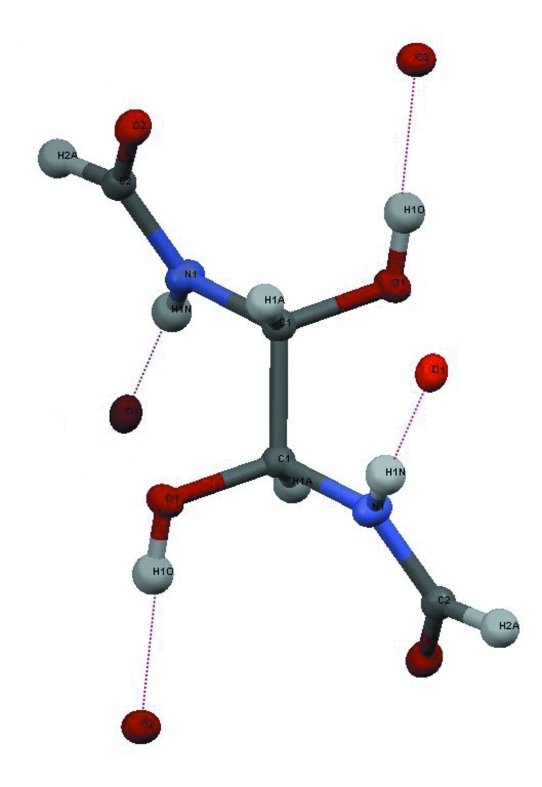
The molecular structure of the title compound, drawn with displacement ellipsoids at the 50% probability level. H atoms are shown as spheres of arbitrary radius.

**Fig. 2.**
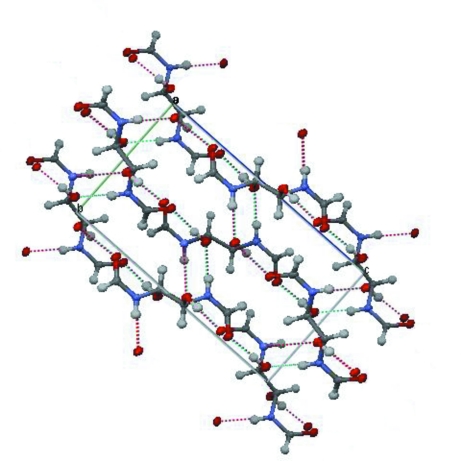
A packing diagram of the title compound. Hydrogen bonds are shown as dashed lines.

## supplementary crystallographic information

### Comment

1,4-Diformyl-2,3,5,6-tetrahydroxypiperazine is an important intermediate (Mitsch, 1965) for the preparation of high energetic materials (Ramakrishnan *et al.* 1990; Vedachalam *et al.* 1991). The title compound, (I), was obtained as an unexpected by-product during synthesis of 1,4-diformyl-2,3,5,6-tetrahydroxypiperazine (Sidney *et al.*, 1965; Ferguson, 1968a,b). In a modified procedure we have synthesized compound (I) in much better yield and present its crystal structure in this communication.

Formally, compound (I) is a derivative of ethane with two hydroxyl and two formyl groups as substitutes of the corresponding H atoms. The asymmetric unit of compound (I) contains one half of the molecule that is completed *via* an inversion centre, leading to a *R*,*S* conformation for the two C atoms (Fig. 1). The bond lengths (Allen *et al.*, 1987) and angles in the molecule are within normal ranges.

In the crystal structure, molecules are arranged in undulated layers parallel to (001). Intermolecular N—H···O and O—H···O hydrogen bonds consolidate this arrangement (Fig. 2 and Table 1).

### Experimental

76 mass parts of glyoxal monohydrate were stirred with 90 parts of formamide at room temperature. Then 6 mass parts of sodium bicarbonate were added. After 3 days, the crude crystalline product was washed with cold methanol and was dried, yielding 84.2 mass parts of 1,4-diformyl-2,3,5,6-tetrahydroxypiperazine (decomposition temperature 463 K). After filtering off the crystals, the aqueous mother liquor was kept at 273 K for 1 day and 2.2 mass parts of 1,2-dihydroxy-1,2-diformamidoethane were obtained (decomposition temperature 408 - 413 K). Crystals suitable for structure determination were grown by recrystallization from dimethyl sulfoxide (DMSO).

### Refinement

H atoms were positioned geometrically, with N—H = 0.88 Å (for NH), O—H = 0.86 Å (for OH) and C—H = 0.95 Å (for the aldehyde group) and and C—H = 1.00 Å (for the aliphatic C atom), and constrained to ride on their parent atoms, with U_iso_(H) = 1.2U_eq_(N, O, C).

### Crystal data

**Table:**

C_4_H_8_N_2_O_4_	*F*_000_ = 312
*M**_r_* = 148.12	*D*_x_ = 1.630 Mg m^−^^3^
Orthorhombic, *P**b**c**a*	Mo *K*α radiation λ = 0.71073 Å
Hall symbol: -P 2ac 2ab	Cell parameters from 854 reflections
*a* = 6.5065 (11) Å	θ = 3–30º
*b* = 7.2634 (12) Å	µ = 0.15 mm^−^^1^
*c* = 12.772 (2) Å	*T* = 120 (2) K
*V* = 603.59 (17) Å^3^	Prism, colourless
*Z* = 4	0.20 × 0.20 × 0.15 mm

### Data collection

**Table:**

Bruker SMART 1000 CCD area-detector diffractometer	662 reflections with *I* > 2σ(*I*)
Radiation source: fine-focus sealed tube	*R*_int_ = 0.031
Monochromator: graphite	θ_max_ = 29.0º
*T* = 120(2) K	θ_min_ = 3.2º
φ and ω scans	*h* = −8→8
Absorption correction: none	*k* = −9→9
5931 measured reflections	*l* = −17→17
796 independent reflections	

### Refinement

**Table:**

Refinement on *F*^2^	Secondary atom site location: difference Fourier map
Least-squares matrix: full	Hydrogen site location: mixed
*R*[*F*^2^ > 2σ(*F*^2^)] = 0.042	H-atom parameters constrained
*wR*(*F*^2^) = 0.107	*w* = 1/[σ^2^(*F*_o_^2^) + (0.0499*P*)^2^ + 0.531*P*] where *P* = (*F*_o_^2^ + 2*F*_c_^2^)/3
*S* = 1.00	(Δ/σ)_max_ < 0.001
796 reflections	Δρ_max_ = 0.41 e Å^−^^3^
46 parameters	Δρ_min_ = −0.24 e Å^−^^3^
Primary atom site location: structure-invariant direct methods	Extinction correction: none

### Special details

**Table:**

Geometry. All e.s.d.'s (except the e.s.d. in the dihedral angle between two l.s. planes) are estimated using the full covariance matrix. The cell e.s.d.'s are taken into account individually in the estimation of e.s.d.'s in distances, angles and torsion angles; correlations between e.s.d.'s in cell parameters are only used when they are defined by crystal symmetry. An approximate (isotropic) treatment of cell e.s.d.'s is used for estimating e.s.d.'s involving l.s. planes.
Refinement. Refinement of *F*^2^ against ALL reflections. The weighted *R*-factor *wR* and goodness of fit *S* are based on *F*^2^, conventional *R*-factors *R* are based on *F*, with *F* set to zero for negative *F*^2^. The threshold expression of *F*^2^ > σ(*F*^2^) is used only for calculating *R*-factors(gt) *etc*. and is not relevant to the choice of reflections for refinement. *R*-factors based on *F*^2^ are statistically about twice as large as those based on *F*, and *R*- factors based on ALL data will be even larger.

### Fractional atomic coordinates and isotropic or equivalent isotropic displacement parameters (Å^2^)

**Table:**

	*x*	*y*	*z*	*U*_iso_*/*U*_eq_	
N1	0.07678 (19)	0.16658 (16)	0.39820 (9)	0.0180 (3)	
H1N	0.1170	0.2562	0.4397	0.022*	
O1	−0.25095 (15)	0.06643 (13)	0.45643 (7)	0.0193 (3)	
H1O	−0.3145	0.0628	0.3970	0.023*	
O2	0.07384 (16)	0.05127 (14)	0.23276 (7)	0.0207 (3)	
C1	−0.0424 (2)	0.01985 (18)	0.44519 (10)	0.0164 (3)	
H1A	−0.0304	−0.0935	0.4012	0.020*	
C2	0.1269 (2)	0.16978 (19)	0.29690 (10)	0.0178 (3)	
H2A	0.2085	0.2694	0.2725	0.021*	

### Atomic displacement parameters (Å^2^)

**Table:**

	*U*^11^	*U*^22^	*U*^33^	*U*^12^	*U*^13^	*U*^23^
N1	0.0226 (6)	0.0168 (5)	0.0147 (5)	−0.0028 (4)	−0.0004 (4)	−0.0002 (4)
O1	0.0165 (5)	0.0254 (5)	0.0160 (4)	0.0025 (4)	−0.0025 (4)	−0.0020 (4)
O2	0.0203 (5)	0.0258 (5)	0.0160 (5)	−0.0010 (4)	0.0020 (4)	−0.0012 (4)
C1	0.0164 (6)	0.0182 (6)	0.0146 (6)	−0.0005 (5)	−0.0003 (5)	0.0004 (5)
C2	0.0168 (6)	0.0196 (6)	0.0170 (6)	0.0025 (5)	0.0007 (5)	0.0035 (5)

### Geometric parameters (Å, °)

**Table:**

N1—C2	1.3344 (17)	O2—C2	1.2374 (17)
N1—C1	1.4483 (17)	C1—C1^i^	1.532 (3)
N1—H1N	0.88	C1—H1A	1.0000
O1—C1	1.4056 (16)	C2—H2A	0.9500
O1—H1O	0.86		
			
C2—N1—C1	123.06 (11)	O1—C1—H1A	109.3
C2—N1—H1N	119.9	N1—C1—H1A	109.3
C1—N1—H1N	117.0	C1^i^—C1—H1A	109.3
C1—O1—H1O	111.4	O2—C2—N1	124.17 (13)
O1—C1—N1	112.47 (11)	O2—C2—H2A	117.9
O1—C1—C1^i^	107.45 (13)	N1—C2—H2A	117.9
N1—C1—C1^i^	108.91 (13)		
			
C2—N1—C1—O1	−99.08 (15)	C1—N1—C2—O2	1.6 (2)
C2—N1—C1—C1^i^	141.93 (15)		

Symmetry codes: (i) −*x*, −*y*, −*z*+1.

### Hydrogen-bond geometry (Å, °)

**Table:**

*D*—H···*A*	*D*—H	H···*A*	*D*···*A*	*D*—H···*A*
N1—H1N···O1^ii^	0.88	2.04	2.9093 (16)	170
O1—H1O···O2^iii^	0.86	1.81	2.6740 (14)	175

Symmetry codes: (ii) *x*+1/2, −*y*+1/2, −*z*+1; (iii) *x*−1/2, *y*, −*z*+1/2.
